# Mass Transfer and Colour Analysis during Vacuum Frying of Colombian Coastal Carimañola

**DOI:** 10.1155/2020/9816204

**Published:** 2020-03-10

**Authors:** Angie D. Caro C, Sandrith P. Sampayo R, Diofanor Acevedo C, Piedad Montero C, Raúl J. Martelo

**Affiliations:** ^1^Faculty of Engineering, Food Engineering Program, Research Group in Agricultural and Agro-Industrial Innovation and Development, University of Cartagena, Colombia; ^2^Faculty of Economic Sciences, Tourism Administration Program, Research Group in Agricultural and Agro-Industrial Innovation and Development, University of Cartagena, Colombia; ^3^Faculty of Engineering, Systems Engineering Program, Research Group in Communications and Informatics Technologies GIMATICA, University of Cartagena, Colombia

## Abstract

This study is aimed at analysing the effect of vacuum frying on the kinetic parameters of mass transfer, the CIE *L*^∗^*a*^∗^*b*^∗^ colour parameters of the Carimañola. For the kinetic analysis, the moisture and oil content were measured by means of an experimental design consisting of two factors: frying time with seven levels (60, 120, 180, 240, 300, 420, and 540 s) and frying temperature with three levels (120, 130, and 140°C). The diffusivity coefficient, the moisture transfer rate, and the oil adsorption rate, with their respective activation energies, were calculated. For the colour analysis, the reflectance technique was used to determine the colour coordinates of the CIE *L*^∗^*a*^∗^*b*^∗^ space, and the general colour change was calculated (Δ*E*). Concerning the kinetics, the increase in temperature and frying time reduced the moisture content, while the oil content decreased with the increase in temperature and increases with frying time. The diffusivity ranged from 1, 238 × 10^−6^ m^2^/s at 120°C to 2, 84 × 10^−6^ m^2^/s at 140°C. The mass transfer coefficients for moisture ranged from 2 × 10^−4^ m/s at 120°C to 4 × 10^−4^ m/s at 140°C. The values of the oil uptake rate were from 0.0022 s^−1^ at 120°C to 0.0018 s^−1^ at 140°C. Finally, the luminosity parameter shows a decrease with the increase in temperature, although the first 240 s shows a rise and then begins to decrease. Vacuum frying allowed Carimañolas to be obtained with a lower oil and moisture content, with an appropriate colouring, eye-catching and visually attractive to consumers.

## 1. Introduction

Cassava (*Manihot esculenta Crantz*) is one of the main foods of the family basket, and in turn, its cultivation is of great importance to the socioeconomic level since this plant can be developed in different climatological conditions. The root is the part of the cassava plant that is mostly consumed by humans, taking advantage of its high nutritional value; in many cases, it is used as raw material for the development of new products for food [1, 2]. However, in many communities, it is only prepared in a homemade way, either boiled, toasted, fried, or converted into intermediate products such as flour and starch; that is to say, a transformation process is not applied that allows obtaining higher added value [3]. Carimañola is a fried by-product made mainly from cassava dough stuffed with meat or cheese, which is cooked in hot oil, giving it sensory characteristics that are attractive to consumers.

Vacuum frying is a healthier frying technique, due to the fact that apart from preserving the sensory qualities of the food, it also reduces the amount of oil uptake [4]. In this operation, the product is placed in a completely closed system and subjected to reduced pressure (subatmospheric), reducing the boiling point of the water and, therefore, the frying temperature. Thus, the water contained in the food is quickly removed when the oil reaches the boiling temperature of the water, so this is a process that requires less time [5].

Transformation processes, such as frying, cause different changes in the food that determine the final characteristics of the product, some of them influenced by the simultaneous heat and mass transfer mechanisms involved in frying [6].

When food is submerged in hot oil, heat transfer occurs by two mechanisms: convection from the oil to the food surface and conduction from the surface to the interior of the food [3]. This heat transfer evaporates part of the water present in the food, which escapes to the surface due to concentration and pressure gradients. Moisture vaporization influences physical and chemical changes, such as starch gelatinization, protein denaturation, darkening, pore and crust formation, and shrinkage/swelling [7, 8].

On the other hand, the absorption of oil during frying depends on a number of factors such as the quality and composition of the oil, the frying time and temperature, the shape and composition of the food, moisture content, surface roughness, and porosity (Manjunatha, Mathews, and Patki, 2019). The absorption of oil in food consists of three parts: *the internal oil*, which is the oil absorbed by the food during frying; *the surface oil absorbed*, which is the oil absorbed by the food immediately after removing it from the hot oil; and *the surface oil*, which is the oil that adheres to the food surface during the cooling stage [9]. It has been proven that during the cooling stage, the food absorbs the greatest amount of oil, because cooling leads to condensation of water vapour in the pores, generating under pressure, and, therefore, oil suction in the pores of the food [10].

The main sensory characteristics of foods are texture, aroma, flavour, and colour, which determine consumer acceptance and perception of quality. Colour is the first attribute evaluated by the consumer, through the sense of sight [11]. The colour of the fried product is an important parameter and must be controlled during the transformation process and is the result of moisture loss, oil migration, and Maillard reaction, which depends on the amount of reducing sugar and amino acids on the surface, temperature, and frying time [12]. The common problem with the processing of Carimañola frying is the formation of dark colour; therefore, during frying, it must be properly controlled.

The traditional Caribbean Carimañola in the frying process undergoes different changes in its sensory, physicochemical, and structural characteristics. The objective of this work was to study the mass transfer through the kinetic parameters and the changes on the chromatic coordinates in the colour space CIE *L*^∗^*a*^∗^*b*^∗^ of the Coastal Carimañola during vacuum frying at different time intervals and temperatures.

## 2. Materials and Methods

### 2.1. Raw Material

The Carimañolas were made with precooked cassava dough of the variety MCOL 2215; this was supplied by a point of sale of fried products located in the municipality of Turbaco (Bolivar) on 14 February 2019; prior to processing the doughs, these were kept refrigerated at a temperature of 12°C. The ground beef, vegetables, and vegetable oil mixture were purchased at a local supermarket in the city of Cartagena de Indias (Colombia) on 13 February 2019. Ground beef and vegetables were refrigerated at 7°C, while oil was stored at room temperature.

### 2.2. Preparation of the Product

Carimañolas were elaborated taking into account the indications given by local fried sellers of the municipality of Turbaco (Bolivar). By-product 60 g of dough and 10 g of ground meat were taken. The portions were kneaded until obtaining circular forms; then, they were pressed in the centre and filled with the meat. Finally, it was proceeded to unite towards the centre to obtain the Carimañolas.

### 2.3. Vacuum Frying Conditions

The cooking process of the Carimañolas was carried out in the GASTROVAC® equipment, whose measures are 40∗26∗46 cm. The maximum capacity of this equipment is 10.5 L with a voltage of 220 V. For the process, a maximum vacuum pressure of 30 kPa was considered, pressure at which the boiling temperature of the water is considered to be 70°C. The temperatures of the frying process were defined using three deltas Δ*T*_1_ = 50°C, Δ*T*_2_ = 60°C, and Δ*T*_3_ = 70°C.

Therefore, the oil temperatures used were 120°C, 130°C, and 140°C, and the frying times used ranged from 180 to 300 s, which were established by preliminary tests. To start the process, Carimañolas were placed in the metal basket that is attached to the lid of the fryer. The fryer was closed, and the equipment was expected to reach the desired pressure. The knob was lowered (submerge the basket in the hot oil). The frying process began; after the frying time, the basket was removed and held with the knob; then, the vacuum chamber was opened, and the hose was disconnected to balance the atmospheric pressure; after this stage, the product is removed from the equipment.

### 2.4. Moisture Loss Kinetics

To analyse the kinetics of moisture loss, an experimental design was made that consisted of two factors: frying time with seven (7) levels (60, 120, 180, 240, 300, 420, and 540 s) and frying temperature with three (3) levels (120, 130, and 140°C) obtaining a total of twenty-one (21) basic combinations. For each one of the treatments after the frying process, moisture was determined at 105°C up to constant weight [13]. Each of these determinations was carried out in triplicate, and the data were expressed as averages on a moisture basis.

### 2.5. Calculation of the Diffusion Coefficients (*D*_*a*_) of Moisture

A first-order kinetic model was chosen for the determination of the diffusivity coefficients of the Carimañolas considering the one-dimensional moisture diffusion described by Fick's second law taking into account the following assumptions: (I) the moisture content is uniform, (II) the geometry is kept constant during the frying process, (III) and the frying temperature is kept constant. Assume the geometry of the Carimañola as a homogeneous cylinder of initial moisture (*M*_o_). Fick's law is described as follows:

(1) $$\begin{array}{c} \frac{\partial x}{\partial t}=D_{a}\frac{\partial^{2}M}{\partial^{2}x}0\leq x\leq r,\;0>t. \end{array}$$

With the initial condition at *t* = 0, the contour conditions at *x* = 0 and the external surface in contact with the oil *x* = +*r*, where *D*_*a*_ is the diffusion coefficient of moisture, *r* is the radius of the cylinder, and *t* is the frying time (s):

(2) $$\begin{array}{c} \text{MR}=\frac{M_{t}+M_{\text{e}}}{M_{\text{o}}+M_{\text{e}}}, \end{array}$$

where *M*_*t*_ is the moisture content at time *t*, *M*_o_ is the initial moisture content, and *M*_e_ is the equilibrium moisture content. Therefore, the analytical solution of the differential equation obtained by the method of separation of variables for cylindrical bodies was applied, used in the first term of the solution series in a similar way to that carried out by [14] in carrots and [15] in peas:

(3) $$\begin{array}{c} \frac{M_{t}+M_{\text{e}}}{M_{\text{o}}+M_{\text{e}}}=\frac{4}{5,783}e^{-\left(5,783D_{a}t/r^{2}\right)}. \end{array}$$

It was assumed that the moisture content is insignificant when equilibrium is reached in the frying process of the Carimañola, so *M*_e_ = 0, rearranging the equation as follows:

(4) $$\begin{array}{c} \frac{M_{t}}{M_{\text{o}}}=\frac{4}{5,783}e^{-\left(5,783D_{a}t/r^{2}\right)}. \end{array}$$

To determine the moisture diffusion coefficient (*D*_*a*_), the linearization of equation (4) was performed by applying logarithms and adjusting it to a straight line *y* = *mt* + *b*:

(5) $$\begin{array}{c} \text{Ln}\left(\frac{M_{t}}{M_{\text{o}}}\right)=-\frac{5,783D_{a}t}{r^{2}}+\text{Ln}\left(\frac{4}{5,783}\right). \end{array}$$

From the graph Ln(*M*_*t*_/*M*_o_)*vs.t*, *D*_*a*_ was estimated from the slope value for the three frying temperatures (120, 130, and 140°C).

The activation energy of the process was then calculated from the diffusion coefficient that fits the Arrhenius equation because of its dependence on temperature [16]:

(6) $$\begin{array}{c} D_{a}=D_{o}e^{-E_{\text{a}}/RT}, \end{array}$$

where *D*_*o*_ is a preexponential factor, *E*_a_ is the activation energy (kJ/mol), *R* is the gas constant (8,314 J/g mol K), and *T* is the absolute temperature (K). By linearizing, the equation was obtained:

(7) $$\begin{array}{c} {\text{Ln}D}_{a}={\text{Ln}D}_{o}-\frac{E_{\text{a}}}{RT}. \end{array}$$

When graphing Ln*D*_*a*_ vs. 1/*T*, the value of the slope with which the value of *E*_a_ was estimated was obtained.

## 3. Calculation of Mass Transfer Coefficient (*k*_*c*_) or Moisture Loss Rate

In order to determine the mass transfer coefficient of the Coastal Carimañola, the first-order Lewis model was used, exposed by [17–20]:

(8) $$\begin{array}{c} \text{MR}=-e^{k_{c}t}, \end{array}$$

where MR is described in equation (8), *k*_*c*_ is the moisture loss rate constant (m/s), and *t* is moisture loss time in frying (s). It then replaces MR, and the equation was linearized to obtain the *k*_*c*_ value of the expression *m* = *k*:

(9) $$\begin{array}{c} \frac{M_{t}}{M_{\text{o}}}=-e^{k_{c}t},\\ \text{Ln}\left(\frac{M_{t}}{M_{\text{o}}}\right)=-k_{c}t. \end{array}$$

### 3.1. Oil Uptake Kinetics

Experimental factorial design of two factors was implemented: temperature (120, 130, and 140°C) and frying time (60, 120, 180, 240, 300, 420, and 540 s), obtaining 21 base treatments. To get the most accurate results, we perform the measurements in triplicate for each treatment. The data collected was presented as the average of the percentage of the gain of dry base oil with its respective standard deviations.

### 3.2. Calculation of Oil Uptake Rate (*k*)

A first-order kinetic model was used to determine the rate of oil uptake [11]:

(10) $$\begin{array}{c} O^{*}=O_{\text{eq}}\left(1-\exp^{-k_{a}}\right), \end{array}$$

where *O*^∗^ is the oil content at time *t* (dry basis) and *O*_eq_ is the oil content at equilibrium (dry basis) when *t* = ∞. Considering that experimentally we obtained the oil content data for frying times, equation (10) was adjusted to a straight line, and when plotting Ln(1 − (*O*^∗^/*O*_eq_)) vs. *t*, we got the slope of the linear section, necessary to calculate the *k* value, which represents the specific oil uptake velocity of the Carimañola (s^−1^). The relationship of oil content variation in equilibrium, *O*_eq_, with frying temperature *T* was evaluated using an Arrhenius type relationship to obtain activation energy [21]:

(11) $$\begin{array}{c} A=A_{0}\exp\left(\frac{-E_{\text{a}}}{RT}\right), \end{array}$$

where *A* is a reaction rate of the model parameters, *A*_0_ is the preexponential factor, *E*_a_ is the activation energy, *R* is the universal gas constant (8,309 J/mol), and *T* is the absolute temperature. With the linearization of equation (11) and the Ln*A*_eq_ vs. 1/*T* graph, we obtained the linear slope, which allowed us to calculate the activation energy. The parameters of the equations were estimated by nonlinear regression, procedures carried out with the Solver tool of Microsoft Excel (Microsoft Corp.), using as objective function the minimization of the defined mean square root (RMS):

(12) $$\begin{array}{c} \text{RMS}=\sqrt{\frac{1}{N}}\overset{N}{\underset{i=1}{\sum }}{\left(\frac{V_{\exp}-V_{\text{fitted}}}{V_{\exp}}\right)}^{2}, \end{array}$$

where *N* is the number of data points, *V*_exp_ is the experimental value, and *V*_fitted_ is the calculated value. Models based on Fick's law will conform to experimental data [22].

### 3.3. Colour Analysis

For colour analysis, the vacuum frying process of the Carimañolas was performed using a Nonrandom Rotating Composite Core Design (DCC-R) consisting of four factorial points, four axial points, and five central points, for a total of 13 experimental treatments (see Table 1).

The colour changes on the outside of the Carimañolas were analysed, using a CR-5 laboratory reflectance colourimeter (Konica Minolta Sensing), with D65 illuminant and a 10° tone angle for the observer. The parameters were evaluated in CIE *L*^∗^*a*^∗^*b*^∗^ space, in terms of luminosity *L*^∗^ (light 100° and dark 0°) and chromaticity *a*^∗^ (red (+) and green (-)) and *b*^∗^ (yellow (+) and blue (-)), where *L*^∗^*a*^∗^*b*^∗^ are the values of each treatment and *L*, *a*, *b* corresponds to the product before frying. The general colour change (Δ*E*) was also calculated for a standard or commercial product using the Euclidean distance, as shown in

(13) $$\begin{array}{c} \Delta E=\sqrt{{\left({\Delta L}^{*}\right)}^{2}+{\left({\Delta a}^{*}\right)}^{2}+{\left({\Delta b}^{*}\right)}^{2}}, \end{array}$$

where Δ*L*^∗^ = difference in light and dark (+ = lighter, - = darker), Δ*a*^∗^ = difference in red and green (+ = redder, - = greener), and Δ*b*^∗^ = difference in yellow and blue (+ = more yellow, - = blue) [23].

### 3.4. Statistical Analyses

A two-way ANOVA and Tukey's HSD multiple comparisons test with a significance level of 5% were used to find statistical differences between the response variable data. A correlation was also made from the *r* Pearson test, with a significance level of 0.01. The data were processed in Statgraphics Centurion 16 (Keygen, U.S.A.).

## 4. Results and Discussion

### 4.1. Moisture Loss Kinetics

**Figure 1:**
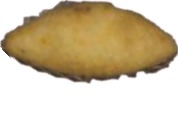
Kinetics of the loss of moisture of the Coastal Carimañolas at different frying times and temperatures.

Figure 1 shows the variation in the moisture content of the Carimañola (as dimensionless moisture *M*_*t*_/*M*_0_) for 120, 130, and 140°C as a function of the frying time under vacuum conditions.

From the kinetic behaviour, the loss of moisture establishes a directly proportional relation with the temperature, and the time of frying was observed; obtaining this way with a time of 540 s for each one of the temperatures (in ascending order), the final content of moisture is 57,23% (±0,79), 55,5% (±0,56), and 51,14% (±0,86); that is to say, to a temperature of 140°C, the Carimañolas presented greater loss of moisture.

This behaviour was also reported by Hase and Linares [3] in the analysis of the water loss kinetics of fried cassava snacks, who state that the increase in temperature produces an increase in the diffusion of moisture from the inside of the food to reach equilibrium after a long frying period. In addition, Figure 1 also shows that high temperatures require less time for the moisture content of the food to evaporate, because the low pressures applied to the process can reduce the boiling point of water and thus frying temperatures. Therefore, when the temperature increases, the water is quickly transformed into steam and then leaves the food through the pores of the same, as indicated by [24] in the vacuum frying of arepas with egg using the same pressure and frying temperatures as in this study. On the other hand, [25] analysed the loss of moisture content of vacuum crisps for a temperature of 125°C reporting a decrease in moisture over time, coinciding with the behaviour analysed for the Coastal Carimañola.

In the frying process, there are other parameters that influence the loss of moisture, within which the shape of the food is highlighted, and the relationship between the size of the product and the surface exposed to the surrounding medium, because if the thickness of the food is greater, there is a smaller specific area and therefore a smaller relative area available to lose water, in addition to the fact that the path that the water particles have to travel towards the external part of the food is longer, so more heat is required to evaporate the water present [26, 27].

### 4.2. Calculation of Diffusion Coefficients (*D*_*a*_) and Mass Transfer Coefficients (*k*_*c*_) or Moisture Loss Rate

**Figure 2:**
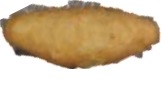
Logarithm of the dimensional moisture concentrations of the Coastal Carimañolas *vs*. the frying time for each temperature.

The moisture diffusion coefficient (*D*_*a*_) was calculated using Fick's second law described for the geometry of a cylinder, from which a *D*_*a*_ estimate of the value of the slopes for temperatures of 120, 130, and 140°C, respectively, was made, as shown in Figure 2, in which we can analyse the rate of loss of moisture of the Carimañolas for each temperature with respect to the time of frying according to the inclination of each one of the slopes; according to this, the straight of 140°C presents a greater inclination and therefore presents a greater rate of loss of moisture, followed by 130 and 120°C; that is to say, the increase of the temperature and the time of frying increases the rate of loss of moisture.

Table 2 shows the moisture diffusivity values for the Coastal Carimañola, at the respective temperatures of 120, 130, and 140°C, obtaining values of 1,238 × 10^−6^, 2,099 × 10^−6^, and 2, 84 × 10^−6^ m^2^/s. The experimental results show that the *D*_*a*_ values are higher for high temperatures.

The effective diffusivity values found for fried Carimañola in vacuum conditions exceed the general range of 10-8 m^2^/s and 10-11 m^2^/s for food dehydration, according to Osorio et al. [28], who in their study when making a comparison of the diffusivity values at different pressures showed greater diffusivity values at decreased pressures and elevated temperatures.

Ortega and Montes [29] in a similar study of fried cassava slices under atmospheric conditions reported diffusivity coefficients of 10.44 × 10^−9^, 17.02 × 10^−9^, and 27.62 × 10^−9^ m^2^/s with temperatures of 140, 160, and 180°C, respectively, evidencing a linear behaviour with the frying temperature, in the same way that was observed in the present study for the Coastal Carimañola but exceeding the magnitude of the values obtained, which can be explained by the difference in pressures used in each study and the shape of the food, in the case of cassava slices which was considered as a flat plate and in the Carimañolas as a cylinder.

On the other side, Alvis et al. [30] reported values of diffusivities similar to the Colombian Coastal Carimañola, of 0.92 × 10^−6^, 1.07 × 10^−6^, and 1.39 × 10^−6^ in pieces of sweet potatoes fried by immersion at temperatures of 150, 170, and 190°C; the authors express that these results exceed the intervals considered by other authors for dehydrated foods attributing these differences to the nature of the product, the temperature, and methods of determination of the frying process.

In starchy foods, the frying process tends to increase diffusivity with porosity and initial moisture content, where the latter two factors establish a relationship with each other, so that food products with a higher degree of porosity have a higher rate of moisture evaporation and therefore a higher coefficient of diffusivity [31]. Carimañolas presented an initial moisture content, before frying, of 65.60% (data not shown) which can be considered an intermediate moisture food, and therefore, the water can be transported by capillary flow and vapour diffusion, which represents an increase in the effective values of diffusivity, facilitating the transport of water through the pores and channels of the food [32].

Vacuum frying causes a hydrodynamic gradient in foods that directly affects their microstructure and consequently their transport properties, as described by Troncoso and Pedreschi [33] in the analysis of the diffusivity of moisture of vacuum-fried potatoes, considering different parameters that may influence it. In addition, they explain that the formation of the external crust or crust in fried food, due to the dehydration process involved in frying, causes a porosity in the crust determined by the frying time, which together with the gelatinization of starch has an effect on the transport properties of water.

The values for *k*_*c*_ for the Carimañolas are shown in the same way in Table 2, varying increasingly with the increase in temperature and frying time, these being 2 × 10^−4^ (120°C), 3 × 10^−4^ (130°C), and 4 × 10^−4^ (140°C) m/s, with *R*^2^ of 0.93, 0.86, and 0.95, respectively, which evidences a good fit of the Lewis kinetic model used to describe the convective coefficient of mass transfer for moisture. [22] found the moisture loss coefficients of yellow pulp cassava slices fried under vacuum and atmospheric conditions using a first-order kinetic model; these authors reported values for vacuum conditions of 0.3177 × 10^−1^, 0.4147 × 10^−1^, and 0.2981 × 10^−1^ m/s for temperatures of 108, 118, and 128°C, respectively, in contrast to the data for atmospheric conditions at 160, 170, and 180°C which were 0.2703 × 10^−1^, 0.2785 × 10^−1^, and 0.2860 × 10^−1^ m/s correspondingly. This shows that vacuum frying allows the food to be processed to obtain the desired characteristics in a period of time similar to that obtained in atmospheric frying. On the other hand, these results obtained under vacuum conditions are greater than those obtained for the Coastal Carimañola.

Ortega and Montes [29] in their research reported mass transfer convective coefficients of 4.41 × 10^−5^, 5.26 × 10^−5^, and 6.03 × 10^−5^ m/s at 140, 160, and 180°C for fried cassava slices by immersion. Similarly, Hase and Linares [3] reported convective coefficients of 2.007 × 10^−2^ (150°C), 2.499 × 10^−2^ (170°C), and 4.068 × 10^−2^ (190°C) for cassava snacks, while [3] reported convective coefficients of 2.007 × 10^−2^ (150°C), 2.499 × 10^−2^ (170°C), and 4.068 × 10^−2^ (190°C) for cassava snacks. In both studies, the authors showed an increase in the convective coefficient of mass transfer with the increase in temperature, a behaviour also observed for the Coastal Carimañola. Tirado et al. [34] determined the dough transfer coefficients of tilapia slices and breadfruit during immersion frying, showing values between 3.31 × 10^−6^ and 9.68 × 10^−6^ m/s for tilapia fillets at temperatures between 130 and 170°C and 6.60 × 10^−9^ and 8.03 × 10^−9^ m/s for breadfruit with the same temperature range, attributing these differences between the coefficients to the pore size of each food, the characteristics of the food matrices, and finally to the method used to determine these coefficients. Other authors used the same kinetic model used in this one for the Coastal Carimañola in the frying by immersion of peas; they reported values between 1.25 × 10^−2^ and 1.94 × 10^−2^ m/s at temperatures between 160 and 200°C [14].

### 4.3. Oil Uptake Kinetics

**Figure 3:**
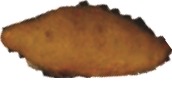
Kinetics of oil uptake during the vacuum frying of the Colombian Coastal Carimañola. The values are averages ± standard deviation.

Oil uptake is a complex phenomenon that has been studied in frying processes. Uptake occurs mainly when the product is removed from the hot oil [33].  Figure 3 shows the oil uptake of Carimañolas fried in vacuum conditions (expressed as the average percentage of oil obtained from repetitions) at temperatures of 120, 130, and 140°C, with respect to a time *t*, which is taken from zero time to 540 s, considering a Carimañola without frying at zero time. In the graph, it is observed that at the same time of frying, as the temperature increases, the fat content decreases; that is to say, the high frying temperatures lead to a lower oil uptake, with results similar to those of [35]. It is also observed that parallel to the increase in time, the uptake of oil increases; the same trend was observed by [32] for vacuum-fried potato slices. This result is in accordance with what was reported by [4], in whose study they evaluated the behaviour of cassava chips in vacuum frying processes. The results found by the authors indicate that the highest fat uptake occurs at the lowest temperature (100°C).

There are several factors that justify the correlation between frying temperature and fat uptake, among which the higher the temperature, the faster the starches gelatinize, and the percentage of free water in the product decreases, creating a barrier for the escape of steam that causes an abrupt expansion in the capillary pores, which as their size increases decrease capillary pressure and oil uptake [36, 37]; it is important that the boiling point of the water is equal to or higher than the gelatinization temperature of Carimañola starch, which has a range of 55-65°C; otherwise, the uptake of oil would not be reduced [22].

When mentioning the expansion in the pores (a phenomenon that occurs mainly in the pressurization stage, i.e., the vacuum is eliminated and the pressure in the pores increases rapidly until it reaches the atmospheric stage), it is normal to think that there is more room for the Carimañola to absorb more oil; however, due to the fact that there is a decrease in pressure, the air spreads much more quickly, which avoids the passage of oil and reduces its uptake [4, 38.

### 4.4. Oil Uptake Rate and Activation Energy

**Figure 4:**
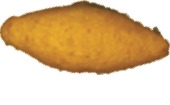
Linear regression of the kinetic model writes the uptake of Carimañolas during vacuum frying.

Figure 4 shows the oil uptake behaviour of the Carimañolas during the vacuum frying process. From the slopes obtained from the linear sections represented in this graph, the oil uptake rate for each temperature was determined, being *m* = −*k*.

Table 3 shows the parameters describing the oil uptake rate and activation energy. The values of the mean square root (%RMS) and the determination coefficient *R*^2^ (close to 1) indicated that there is a good fit of the regression model, with the values observed, i.e., that the regression line was significantly close to these values; these results are similar to those reported by [16], in whose study *R*^2^ corresponding to the temperature of 140°C exceeds the value of 0.95.

In the table, *k*-values indicate a tendency for the oil uptake rate to decrease with increasing temperatures. This trend is consistent with that reported by [22] who studied the kinetics of the uptake of oil from fried cassava slices under vacuum conditions; the values reported were 0.0384, 0.0340, and 0.0324 s^−1^ for temperatures of 108, 118, and 128°C. The values of this research are comparatively higher than those found in Carimañolas, a difference that could be attributed to the fact that Carimañola is not composed only of cassava and its thickness. The same trend was also observed in the study by [39] about the determination of the coefficients of thermal diffusivity of moisture and oil in breadfruit, whose values were 0.001, 0.001, and 0.0009 s^−1^ at temperatures of 120, 130, and 140°C. The same trend was observed in the study by [39] about the determination of the coefficients of thermal diffusivity of moisture and oil in breadfruit, whose values were 0.001, 0.001, and 0.0009 s^−1^ at temperatures of 120, 130, and 140°C. It should be noted that, although there is a tendency to increase the *k* parameter with temperature, it does not have a significant effect on the uptake of oil [33].

The values of *A*_eq_ represent the maximum amount of oil that the Carimañolas could absorb; in Table 3, it can be observed that this parameter increases as the frying temperature is reduced; these results agree with those reported by [35, 39].

Thermodynamically, the activation energy is the energy required by water molecules for their migration or movement within a product; it is considered that this comes initially from the thermal energy supplied by the oil and is independent of moisture content. *E*_a_ was calculated using the slope indicated in Table 3 (*m* = 1439.2), being *E*_a_ = *m*∗*R* (“*m*” as the slope and “*R*” as the universal gas constant); in Table 3, it is observed that the value obtained is -11.96 kJ/mol. This result is similar to the one reported by [39], who pointed out that for temperatures of 120, 130, and 140°C, the value of *E*_a_ was -24.78 kJ/mol; the authors stressed that the negative value is due to the fact that the oil uptake index decreases with the increase in temperature, as occurring in the present study. According to [28], another factor that justifies the value of *E*_a_ being so low is that it depends on the vacuum pressure of the medium, since the greater the vacuum, the less *E*_a_ is required to start the process.

### 4.5. Colour Analysis

Table 4 shows that time caused a greater effect than temperature on parameter *L*^∗^, evidencing that this one decreased as the temperature increased; on the other hand, if the time is analysed, the luminosity increased until the first 240 s; from then on, it decreased linearly. The *L*^∗^ parameter did not differ significantly at lower temperatures (115.85 at 120°C) or when this factor increased. Parameter *a*^∗^ did not show significant differences at shorter frying times; the highest values were observed at temperatures of 130°C. Mariscal and Bouchon [40] reported that there were no significant differences in the luminosity of the precooked and vacuum-fried apple slices at 10 minutes of frying. They also reported that the rate of reduction of values for parameter *L*^∗^ at higher frying times (i.e., 10 to 12 min) was slower compared to the rate observed at a lower rate. Since luminosity is a very important colour quality at lower frying temperatures (especially under vacuum conditions), with a lower boiling point of water, they are preferable to preserve the luminosity and therefore the attractiveness of fried products. In contrast, redness is an undesirable quality factor in fried foods [41]; the increase in redness shows an increase in crust development, resulting in lower acceptability. Increased redness for all frying treatments may mean that all treatments (e.g., potatoes) experience an increase in gold with increasing frying temperature and time. This could be due to Maillard's reaction resulting from the use of available reducing sugars.

The colour development of the product during frying depends on the drying rate (moisture loss), oil uptake, and heat transfer coefficient in the different frying stages. The brightness value is a critical parameter in the frying process and is considered as a primary quality factor evaluated by the consumer. Results of low luminance values are dark in colour and are formed due to nonenzymatic darkening reactions. Brightness values for potato chips greater than 60 were termed as excellent, 56-60 as acceptable, and less than 50-55 as slightly acceptable [41, 42] confirmed for Gethi frying that redness values *a*^∗^ increased significantly (*p* < 0.05) with increasing frying time and temperature. The redness value was 2.48, and after 15 minutes of frying, it increased to 5.77, 7.52, 12.71, and 18.53 at 120, 140, 160, and 180°C frying temperature, respectively. The increase in Hunter redness (*a*) value may be due to moisture loss, oil impact, and the formation of Maillard reaction products during frying of Gethi strips. Similar results were reported in the case of frying potato discs by immersion [43]. Redness values were significantly higher for atmospheric pressure chips than for vacuum chips due to a marked increase in Maillard reaction products [38].

The highest colour differences during the frying of Carimañolas were evident at lower temperatures (115.858°C) and 180 s. High temperatures and longer times obtained smaller differences with the standard, which could be considered an option to recommend the frying of the Carimañola to these conditions. The increase in the magnitude of total colour difference values could be attributed to high temperature and low moisture content, which initiated nonenzymatic browning, such as Maillard's reaction and sugar caramelization. Mariscal and Bouchon [40] observed that vacuum potato chips had the smallest overall colour change compared to atmospheric potato chips. This implies that the colour difference between these two types of frying may be due to the act that the processing conditions are also a reflection of the degree of degradation of total carotene, which could further establish that vacuum frying has the highest levels of total carotene retention.

## 5. Conclusions

The vacuum frying process of the Colombian Caribbean Carimañola allows us to obtain a product with excellent bromatological and nutritional characteristics, as well as a lower oil content. Kinetic and transfer parameters showed that temperature and frying time factors have a significant effect on Carimañola characteristics and composition. The increase in temperature and frying time indicates a reduction in moisture content, while oil uptake decreasing with increasing temperature increases with frying time. In addition, high temperatures also cause darker colourations. Therefore, vacuum frying is a viable alternative for the processing of Colombian Coastal Carimañola.

## Figures and Tables

**Table 1:** Central composite rotational design matrix (DCC-R) with axial points and coded central points: vacuum frying.

Variables	Encoder symbol	Range and levels				
		*α*_1_ = −1.4241 (axial)	-1 (lowly)	0 (central)	+1 (louder)	*α*_2_ = +1.4241 (axial)
Temperature (°C)	*X*_1_	115,858	120	130	140	144,142
Time (s)	*X*_2_	155,26	180	240	300	324,85

**Table 2:** Parameters of mass transfer of vacuum-fried Coastal Carimañolas: diffusivity and convective moisture coefficient.

*T* (°C)	*D*_*a*_ (m^2^/s)	*k*_*c*_ (m/s)	*E*_a_	*R* ^2^
120	1,238 × 10^−6^	2 × 10^−4^	56,11 kJ/mol	0,94
130	2,099 × 10^−6^	3 × 10^−4^		0,86
140	2, 84 × 10^−6^	4 × 10^−4^		0,95

**Table 3:** Parameters describing the oil uptake kinetics of the Coastal Carimañolas and the activation energy with Arrhenius type adjustment.

	Oil uptake rate			Calculation of the activation energy					
*T* (°C)	*k* (s^−1^)	*R* ^2^	%RMS	1/*T* (K)	*A*_eq_	Ln*A*_eq_	Pending (m)	*R* (kJ mol/K)	*E*_a_
120	0.0022	0.963	0.51	0.00254	23.390	3.152	1439.2	8.31	-11.96
130	0.002	0.955	3.00	0.00248	20.451	3.018			
140	0.0018	0.961	1.02	0.00242	19.606	2.976			

**Table 4:** Colour changes of the Carimañolas as a function of time and temperature.

Vacuum frying		
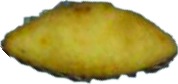	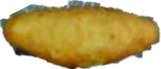	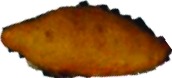
*T1 (130°C-324.853 s)*	*T2 (120°C-300 s)*	*T3 (140°C-180 s)*
*L* ^∗^ = 63.85 ± 2.27^b^	*L* ^∗^ = 68.12 ± 2.61^bcdef^	*L* ^∗^ = 70.14 ± 2.62^cdef^
*a* ^∗^ = 10.07 ± 1.62^f^	*a* ^∗^ = 8.41 ± 0.63^def^	*a* ^∗^ = 6.25 ± 0.60^ab^
*b* ^∗^ = 38.11 ± 1.31^a^	*b* ^∗^ = 39.85 ± 1.84^a^	*b* ^∗^ = 37.61 ± 2.61^a^
Δ*E* = 7.51	Δ*E* = 12.01	Δ*E* = 14.02

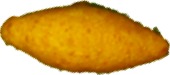	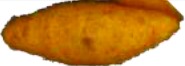	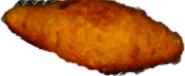
*T4 (130°C-240 s)*	*T5 (140°C-300 s)*	*T6 (115.858°C-240 s)*
*L* ^∗^ = 67.01 ± 2.12^ef^	*L* ^∗^ = 66.02 ± 0.35^bc^	*L* ^∗^ = 72.34 ± 2.16^f^
*a* ^∗^ = 8.18 ± 1.39^a^	*a* ^∗^ = 7.42 ± 0.37^bcd^	*a* ^∗^ = 6.49 ± 0.90^abc^
*b* ^∗^ = 37.41 ± 0.83^a^	*b* ^∗^ = 36.89 ± 2.1^a^	*b* ^∗^ = 37.8 ± 0.82^a^
Δ*E* = 10.62	Δ*E* = 9.75	Δ*E* = 16.14

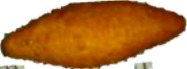	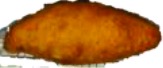	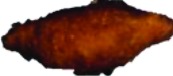
*T7 (120°C-180 s)*	*T8 (130°C-240 s)*	*T9 (130°C-155.147 s)*
*L* ^∗^ = 71.67 ± 4.33^ef^	*L* ^∗^ = 71.37 ± 3.66^bcd^	*L* ^∗^ = 69.58 ± 2.81^cdef^
*a* ^∗^ = 7.17 ± 2.33^bcd^	*a* ^∗^ = 5.84 ± 0.72^ab^	*a* ^∗^ = 6.68 ± 0.30^abcd^
*b* ^∗^ = 38.92 ± 2.05^a^	*b* ^∗^ = 37.59 ± 2.09^a^	*b* ^∗^ = 37.28 ± 1.64^a^
Δ*E* = 15.47	Δ*E* = 15.31	Δ*E* = 13.38

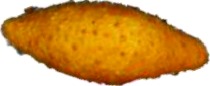	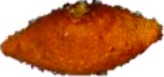	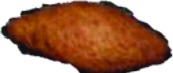
*T10 (130°C-240 s)*	*T11 (130°C-240 s)*	*T12 (144,142°C-240 s)*
*L* ^∗^ = 54.8 ± 2.41^a^	*L* ^∗^ = 63.67 ± 4.42^def^	*L* ^∗^ = 67.56 ± 1.22^bcd^
*a* ^∗^ = 13.27 ± 0.87^cde^	*a* ^∗^ = 9.31 ± 0.44^g^	*a* ^∗^ = 8.26 ± 0.69^de^
*b* ^∗^ = 37.59 ± 1.51^a^	*b* ^∗^ = 37.88 ± 2.06^a^	*b* ^∗^ = 37.66 ± 2.39^a^
Δ*E* = 4.37	Δ*E* = 7.26	Δ*E* = 11.17

	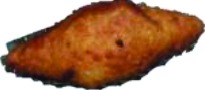	
	*T13 (130°C-240 s)*	
	*L* ^∗^ = 72.08 ± 1.46^b^	
	*a* ^∗^ = 5.31 ± 0.62^ef^	
	*b* ^∗^ = 37.29 ± 2.06^a^	
	Δ*E* = 16.12	

## Data Availability

The data used as references in this study have been indicated in the part of the reference repository (name of authors, year of publication, title of the document, journal and number of pages).

## References

[1] Villada D., Villada H., Mosquera A. (2009). Evaluación del efecto de la deshidratación osmótica y fritura en dos variedades de yuca (*Manihot esculenta crantz*) en la producción de chips. *Dyna*.

[2] Duran P., Rojas M. (2013). Propuesta metodológica para la evaluación de las características fisicoquímicas de dos variedades de yuca (*Manihot esculenta Crantz*), utilizadas como materia prima para la preparación de hojuelas fritas. *Scientia et Technica*.

[3] Hase S. L., Linares A. R. (2018). Cinética de pérdida de agua y ganancia de aceite de snacks fritos de mandioca. *Revista de Ciencia y Tecnología*.

[4] Urbano A. M., García P., Martínez J. (2012). *Evaluación del comportamiento de yuca (Manihot esculeta Cranz) en el proceso de fritura a vacío de chips*.

[5] Villamizar R., Quiceno M., Germán G. (2016). Physicochemical and sensory valuation of plantain snacks (*AAB, Musa paradisiaca sp*.) in vacuum frying. *Agronomía Colombiana*.

[6] Moreira R. (2001). Deep-fat frying of foods. *Food Processing Operations Modeling: Design and Analysis*.

[7] Sampayo-Rodriguez S., Montero P. M., Paternina-Sierra K. (2018). Weight losses determination of coastal carimanola during vacuum frying. *Contemporary Engineering Sciences*.

[8] Yagua C. V., Moreira R. G. (2011). Physical and thermal properties of potato chips during vacuum frying. *Journal of Food Engineering*.

[9] van Koerten K. N., Schutyser M. A. I., Somsen D., Boom R. M. (2015). Crust morphology and crispness development during deep-fat frying of potato. *Food Research International*.

[10] Vitrac O., Trystram G., Raoult-Wack A. L. (2000). Deep-fat frying of food: heat and mass transfer, transformations and reactions inside the frying material. *European Journal of Lipid Science and Technology*.

[11] Hernandez E. (2005). *Evaluación Sensorial*.

[12] Krokida M. K., Oreopoulou V., Maroulis Z. B., Marinos-Kouris D. (2001). Colour changes during deep fat frying. *Journal of Food Engineering*.

[13] AOAC (2005). *Association of Official Analytical Chemists, Official Methods of Analysis of AOAC International*.

[14] Melquíades Y. I., López C., Rosas M. E. (2009). Estudio de la cinética de rehidratación de zanahoria (*Daucus Carota*) deshidratadas. *Información tecnológica*.

[15] Barrios Barrios L., Osorio Mora O., Ceron Cardenas A. F. (2016). Estudio de las cinéticas de pérdida de agua y absorción de aceite durante la fritura de arveja (*Pisum sativum* L.). *Acta Agronómica*.

[16] Alvis A., Vélez C., Arrázola G. (2010). Efecto de las condiciones de freído sobre la pérdida de humedad y ganancia de aceite en trozos de ñame (*Dioscorea alata*). *Ingeniería e Investigación*.

[17] Aykın-Dinçer E., Erbaş M. (2018). Drying kinetics, adsorption isotherms and quality characteristics of vacuum-dried beef slices with different salt contents. *Meat Science*.

[18] Ricce C., Rojas M. L., Miano A. C., Siche R., Augusto P. E. D. (2016). Ultrasound pre-treatment enhances the carrot drying and rehydration. *Food Research International*.

[19] Vijayan S., Arjunan T. V., Kumar A. (2016). Mathematical modeling and performance analysis of thin layer drying of bitter gourd in sensible storage based indirect solar dryer. *Innovative Food Science & Emerging Technologies*.

[20] da Rocha R. P., Melo E. d. C., Corbín J. B., Berbert P. A., Donzeles S. M. L., Tabar J. A. (2012). Cinética del secado de tomillo. *Revista Brasileira de Engenharia Agrícola e Ambiental*.

[21] Pedreschi F., Hernandez P., Figueroa C., Moyano P. (2005). Modeling water loss during frying of potato slices. *International Journal of Food Properties*.

[22] Oyedeji A. B., Sobukola O. P., Henshaw F. O., Adegunwa M. O., Sanni L. O., Tomlins K. I. (2016). Kinetics of mass transfer during deep fat frying of yellow fleshed cassava root slices. *Heat and Mass Transfer*.

[23] Konica Minolta (2007). *Precise Color Communication: Color Control from Perception to Instrumentation*.

[24] Torres J. D., Acevedo D., Montero P. M. (2017). Efectos de la fritura al vacío en los atributos de calidad de arepa con huevo. *Información tecnológica*.

[25] Belkova B., Hradecky J., Hurkova K., Forstova V., Vaclavik L., Hajslova J. (2018). Impact of vacuum frying on quality of potato crisps and frying oil. *Food Chemistry*.

[26] Bravo J. (2008). *Contribución al estudio de la fritura al vacío: deshidratación de rodajas de manzana, [Ph.D. thesis]*.

[27] Gómez S., Martínez J., García P. (2013). *Efecto de las condiciones de fritura a vacío en el procesado de chips de kiwi (Actidinia chinensis)*.

[28] Osorio O., Rodríguez G., CasteNanos F., Chávez A. (2016). Procesamiento de arvejas (*Pisum sativum* L.). Parte 3: Cinética de pérdida de agua en chips de arveja en condiciones de fritura convencional y a vacío. *Información tecnológica*.

[29] Ortega F. A., Montes E. J. (2014). Parámetros cinéticos de transferencia de masa durante el freído por inmersión de rodajas de yuca (*Manihot esculenta Crantz*). *Ingeniería y Competitividad*.

[30] Alvis A., González A., Arrázola G. (2015). Efecto del recubrimiento comestible en las propiedades de trozos de batata (*Ipomoea Batatas* Lam) fritos por inmersión: Parte 2: Propiedades termofísicas y de transporte. *Información tecnológica*.

[31] Torres-Gonzalez J. D., Alvis-Bermudez A., Gallo-Garcia L. A., Acevedo-Correa D., Castellanos-Galeano F., Bouchon-Aguirre P. (2018). Effect of deep fat frying on the mass transfer and color changes of arepa con huevo. *Indian Journal of Science and Technology*.

[32] Saravacos G. D., Maroulis Z. B. (2001). *Transport Properties of Foods*.

[33] Troncoso E., Pedreschi F. (2009). Modeling water loss and oil uptake during vacuum frying of pre-treated potato slices. *LWT - Food Science and Technology*.

[34] Tirado D. F., Acevedo D., Montero P. M. (2015). Transferencia de calor y materia durante el proceso de freído de alimentos: tilapia (*Oreochromis niloticus*) y fruta de pan (*Artocarpus communis*). *Información tecnológica*.

[35] Moyano P. C., Pedreschi F. (2006). Kinetics of oil uptake during frying of potato slices: Effect of pre- treatments. *LWT - Food Science and Technology*.

[36] Ziaiifar A. M., Achir N., Courtois F., Trezzani I., Trystram G. (2008). Review of mechanisms, conditions, and factors involved in the oil uptake phenomenon during the deep-fat frying process. *International Journal of Food Science & Technology*.

[37] Moreira R. G., Da Silva P. F., Gomes C. (2009). The effect of a de-oiling mechanism on the production of high quality vacuum fried potato chips. *Journal of Food Engineering*.

[38] Garayo J., Moreira R. (2002). Vacuum frying of potato chips. *Journal of Food Engineering*.

[39] Correa D. A., Gallo-García L. A., González-Morelo K. J. (2017). Determination of the moisture and oil thermal diffusivity coefficients in breadfruit (*Artocarpus altilis*) during vacuum frying. *International Journal of Engineering and Technology*.

[40] Mariscal M., Bouchon P. (2008). Comparison between atmospheric and vacuum frying of apple slices. *Food Chemistry*.

[41] Pangloli P., Melton S. L., Collins J. L., Penfield M. P., Saxton A. M. (2002). Flavor and storage stability of potato chips fried in cottonseed and sunflower oils and palm olein/sunflower oil blends. *Journal of Food Science*.

[42] Manjunatha S. S., Ravi N., Negi P. S., Raju P. S., Bawa A. S. (2014). Kinetics of moisture loss and oil uptake during deep fat frying of Gethi (*Dioscorea kamoonensis Kunth*) strips. *Journal of Food Science and Technology*.

[43] Sahin S. (2000). Effects of frying parameters on the colour development of fried potatoes. *European Food Research and Technology*.

